# Role of the Cellular Prion Protein in Oligodendrocyte Precursor Cell Proliferation and Differentiation in the Developing and Adult Mouse CNS

**DOI:** 10.1371/journal.pone.0033872

**Published:** 2012-04-18

**Authors:** Ana Bribián, Xavier Fontana, Franc Llorens, Rosalina Gavín, Manuel Reina, José Manuel García-Verdugo, Juan María Torres, Fernando de Castro, José Antonio del Río

**Affiliations:** 1 Molecular and Cellular Neurobiotechnology, Catalonian Institute for Bioengineering (IBEC), Parc Científic de Barcelona, Barcelona, Spain; 2 Department of Cell Biology, Universitat de Barcelona, Barcelona, Spain; 3 Centro de Investigación Biomédica en Red sobre Enfermedades Neurodegenerativas (CIBERNED), Barcelona, Spain; 4 Laboratorio de Neurobiología Comparada, Instituto Cabanillas de Biodiversidad y Biología Evolutiva, Universidad de Valencia, Valencia, Spain; 5 Centro de Investigación en Sanidad Animal (CISA-INIA), Madrid, Spain; 6 GNDe-Grupo de Neurobiología del Desarrollo, Unidad de Neurología Experimental, Hospital Nacional de Parapléjicos, Toledo, Spain; 7 Instituto Cajal-CSIC, Madrid, Spain; Sanford-Burnham Medical Research Institute, United States of America

## Abstract

There are numerous studies describing the signaling mechanisms that mediate oligodendrocyte precursor cell (OPC) proliferation and differentiation, although the contribution of the cellular prion protein (PrP^c^) to this process remains unclear. PrP^c^ is a glycosyl-phosphatidylinositol (GPI)-anchored glycoprotein involved in diverse cellular processes during the development and maturation of the mammalian central nervous system (CNS). Here we describe how PrP^c^ influences oligodendrocyte proliferation in the developing and adult CNS. OPCs that lack PrP^c^ proliferate more vigorously at the expense of a delay in differentiation, which correlates with changes in the expression of oligodendrocyte lineage markers. In addition, numerous NG2-positive cells were observed in cortical regions of adult PrP^c^ knockout mice, although no significant changes in myelination can be seen, probably due to the death of surplus cells.

## Introduction

Oligodendrocyte maturation and differentiation is a well-orchestrated process that has been studied in detail in isolated oligodendrocyte precursor cells (OPCs) in culture, where the proliferation and differentiation of OPCs is controlled by a well-defined sequence of events (see for example, [1], [2]). In recent years, numerous studies have sought to identify new factors that regulate OPC proliferation and differentiation (see [3], [4], [5] for reviews). During development, immature proliferative oligodendrocytes are characterized by the expression of the chondroitin sulphate proteoglycan, NG2, together with other markers, such as the platelet-derived growth factor receptor alpha (PDGFr-α) and the cell surface ganglioside A2B5 antigen [5], [6], [7], [8], [9], [10]. During maturation, oligodendrocytes sequentially express markers such as adenomatous polyposis coli (APC-CC1) and 2',3'-cyclic nucleotide 3'-phosphodiesterase (CNPase), as well as markers of myelinating oligodendrocytes, such as myelin basic protein (MBP) and myelin-associated glycoprotein (MAG). Other antigens of the oligodendrocyte lineage, such as the helix-loop-helix transcription factors *Olig2* and *Sox10*, are expressed in immature as well as mature myelinating oligodendrocytes [11]. In the adult brain, the OPCs that persist are considered to be a putative reservoir of mature oligodendrocytes. These cells proliferate and differentiate into myelinating oligodendrocytes in order to maintain myelin in both the healthy and injured brain [9], [12], [13], [14], [15], [16], [17], [18], [19]. Adult OPCs in the neuronal parenchyma are NG2-positive (see [20] for review) and they are considered to be cycling cells with the capacity to differentiate into mature oligodendrocytes, as well as protoplasmic astrocytes [15] and neurons [13], [21].

The process of myelination is influenced by many factors, including EGFr-mediated signaling [22]. EGFr signaling is a complex process that is dependent upon trans-activation by other membrane-associated proteins or receptors (*e.g.,* GPCRs) [23]. Molecules or receptors previously thought to be unrelated to EGFr-mediated signaling have recently been characterized as putative modulators of EGFr pathways. One example in the CNS is the cellular prion protein (PrP^c^), a glycosyl phosphatidyl inositol (GPI)-anchored cell surface protein encoded by the *Prnp* gene [24], [25], [26], [27]. Clustering of PrP^c^ at the cell surface has been shown to modulate EGFr activity in GT1-7 cells [28], and while the developmental functions of PrP^c^ remain to be fully determined, PrP^c^ may help maintain myelin in both the CNS and the peripheral nervous system (PNS) [29]. However, a putative link between PrP^c^ and OPC proliferation or oligodendrocyte differentiation in the CNS has not yet been fully determined.

Accordingly, we have analyzed how PrP^c^ might influence the proliferation and differentiation of embryonic OPCs and of adult NG2 expressing cells. We isolated OPCs from diverse origins and developmental stages, and analyzed their distribution in the forebrain of adult *Prnp^0/0^* and *Prnp^+/+^* mice. The absence of PrP^c^ increased the number of undifferentiated oligodendrocytes and delayed the expression of differentiation markers *in vitro* (*e.g., Sox17, cdk2*, *APC*, *CNPase*). In accordance with these *in vitro* findings, the large numbers of cells expressing Olig2 and NG2 were evident in the cortical parenchyma of developing and adult mice. Surprisingly, the increase in the number of NG2 expressing cells was not correlated with alterations in myelination, suggesting that compensatory mechanisms may have offset this effect. Indeed, the number of BrdU-labeled OPCs in the *Prnp^0/0^* cortex two weeks after pulse labeling decreased significantly to wild-type level. This decrease was correlated with the appearance of TUNEL labeling in the NG2 expressing cells, suggesting that surplus OPCs are eliminated by cell death in the adult *Prnp^0/0^* cortex.

## Methods

### Mice

*Prnp^0/0^* Zürich-1 mice were purchased from EMMA (Monterotondo, Italy) and they carried approximately 46.8% C57BL/6J microsatellite markers (Charles River Laboratories). To avoid putative background-related differences, we backcrossed our *Prnp^0/0^* mice with C57BL/6J mice over several generations. All experiments were carried out using littermates derived from selected heterozygous (*Prnp^0/+^*) parents after backcrossing (50 littermates: 34 adult mice and 16 newborn mice). The presence of C57BL/6J markers in all the mouse phenotypes used in the present study was determined by the Genetic testing service at Charles River Laboratories, analyzing 110 microsatellite markers at approximately 15 cM intervals across the 19 autosomes and the X chromosome. This analysis distinguishes between 129 microsatellite markers ranging from 92 to 95% of C57BL/6J. For genotyping, the following specific primers for *Prnp^0/0^* (Zürich I) were designed [30]: P10-new: 5′-cataatcagtggaacaagccc-3′; P4-new: 5′-gctacaggtggataacccctc-3′; P3-new: 5′-gccttctatcgccttcttgac-3′. PCR was performed over 40 cycles: 4 minutes at 95°C; 4 minutes at 62°C and 1 minute at 72°C; followed by a final extension for 5 minutes at 72°C. We did not analyze the behavior of OPCs in mice overexpressing PrP^c^ (*e.g.,* Tga20), as differences in PrP^c^ expression have been reported in these animals when compared to wild type mice [31], [32]. All studies were performed under the guidelines and protocols of the Ethical Committee for Animal Experimentation (CEEA) at the University of Barcelona, and the protocol for the use of animals in this study was reviewed and approved by the CEEA at the University of Barcelona (CEEA approval# 115/11).

### Antibodies

The following antibodies were used to detect OPCs: rabbit anti-NG2 and anti-Olig2 (1∶200: Chemicon, Temecula, CA, USA), mouse monoclonal anti-A2B5 (1∶10, mAb 4D4: Developmental Studies Hybridoma Bank-DSHB, University of Iowa, USA), and anti-Nestin (1∶1000: Chemicon). To detect mature oligodendrocytes and myelin we used a rabbit antiserum against CNPase (1∶200: Thermo Scientific, Fremont, USA) or MAG (1∶1000: Santa Cruz biotechnology, Santa Cruz, USA), or a mouse monoclonal against MBP (1∶2000: Chemicon). To detected astrocytes and neurons, we used a mouse monoclonal against GFAP (1∶500: Dako Glostrup, Denmark) and NeuN (1∶50: Chemicon), respectively. Proliferating cells were detected using a rat monoclonal antibody raised against BrdU (1∶50: Harlan Sera-Lab, Loughborough, England). To probe western blots, a mouse monoclonal antibody against actin (1∶10000) or tubulin (1∶1000; Chemicon) were also used. Two different mouse monoclonal antibodies were used to detect PrP^c^: SAF61 (1∶1000: Spi-Bio & Cayman Chemical, Massy Cedex, France) and 6H4 (1∶200: Prionics, Schlieren, Switzerland).

### Embryonic Optic Nerve Cultures

The embryonic optic nerves (ONs) from E16.5 embryos were dissected out and cultured as described previously [33], [34]. Briefly, ON explants were placed in three-dimensional gels of rat tail-derived collagen and cultured in Bottenstein-Sato medium supplemented with FGF-2 (20 ng/ml: R&D Systems, Minneapolis, USA) at 37°C, in an atmosphere of 5% CO_2_ and at 95% humidity. After 3 days *in vitro* (DIV), genotypically identified cultures were fixed with 4% paraformaldehyde (PFA) in 0.1 M phosphate buffered saline (PBS, pH 7.4). The number of cells migrating out of the explants was counted and the maximum distance migrated with respect to the center of the ON explants was determined. Cell proliferation was assessed by BrdU incorporation (50 µM: Sigma-Aldrich, Poole Dorset, UK) added to the medium for 6 hours (from 42–48 hours post-culture) as described previously [35]. The medium was then removed and the cultures were fixed as described above.

### Cortical OPC Purification

Primary cultures were prepared from *Prnp^0/0^* and *Prnp^+/+^* mouse pups (P0–P2) as described previously [36], [37]. Cortical tissue was dissected out and digested at 37°C with trypsin and DNAse (Sigma-Aldrich) in HBSS (without Ca^2+^ and Mg^2+^). After centrifugation, the cells were resuspended in 10 ml of DMEM containing 10% fetal bovine serum and antibiotics (DMEM medium). This suspension was then filtered through 100 µm filters and the cells were seeded in 75 ml flasks and 6-well culture plates previously coated with poly-L-ornithine (Sigma-Aldrich), changing the medium every three days. To obtain differentiated cells, once 80–90% confluence was reached the medium was switched to serum free DMEM medium supplemented with T3, T4, putresceine, progesterone and sodium selenite (SFM; all from Sigma-Aldrich). These cells were then maintained for 5 DIV to allow them to differentiate into mature oligodendrocytes. As cell death in *Prnp^0/0^*-cultured cells augments after serum removal [38], [39], OPCs were cultured over a feed layer of astrocytes for 5 days (mixed cultures). Cultures were then processed to obtain purified oligodendrocytes. Thus, cultures were shaken at 250 r.p.m. overnight at 37°C, and the medium was then filtered through 40 µm filters and centrifuged at 800 r.p.m for 5 minutes. The pellet containing the OPCs was analyzed by RT-qPCR or Western blotting. However, both *Prnp^0/0^* and *Prnp^+/+^* derived OPCs were cultured in parallel.

To analyze a putative influence of astrocytes in the behavior of OPCs, when the cultures reached 80–90% confluence in DMEM medium they were shaken to purify the OPCs as described above (isolated cultures). The isolated OPCs were then cultured in SFM for additional 5 DIV and their differentiation, survival and proliferation in the absence of astrocytes was analyzed. In addition, we also obtained RNA for RT-qPCR from these purified cells as described above for the mixed cultures. Lastly, freshly shaked OPCs isolated cultures from *Prnp^+/+^* and *Prnp^0/0^* mixed cultures were treated with different concentrations of conditioned medium (DMEM) derived from cultured astrocytes from the opposite genotype. After 5 DIV, treated cultures were fixed, double labeled using CNPase and Olig2 antibodies and quantified.

### Neurosphere Isolation and Differentiation *in vitro*

To prepare neurospheres, *Prnp^0/0^* and *Prnp^+/+^* P5 pups were anaesthetized by hypothermia and their brains were removed from the skull aseptically. The subventricular zone of the lateral ventricle was dissected in cold HBSS (without Ca^2+^ or Mg^2+^), and the cells were cultured and differentiated as described previously [40], in culture medium supplemented with B27, antibiotics, FGF-2 and EGF (20 ng/ml, Sigma-Aldrich: unless otherwise indicated, all culture media and supplements were purchased from GIBCO Life Technologies, Merelbeke, Belgium). Growing spheres were mechanically dissociated each week and plated in fresh medium (1 passage/week). In the differentiation experiments, after mitogen withdrawal neurospheres were grown on poly-L-ornithine and laminin (Sigma-Aldrich) coated coverslips (12 mm) for 7 days in serum-free medium (DMEM containing glutamine, B27 and antibiotics). After differentiation, the cultures were fixed with 2% paraformaldehyde for 1 hour at 4°C and they were then processed for immunocytochemistry with Alexa-Fluor 488 and 546 tagged secondary antibodies (Molecular Probes, Eugene, USA). After rinsing, the cell nuclei were counterstained for 10 minutes with DAPI (1 µM in 0.1 M PBS) and the cells were mounted in Fluoromount^TM^ (Vector Labs, Burlingame, USA). The cells were examined on an Olympus BX61 or an Olympus Fluoview SV 500 confocal microscope, obtaining images in sequential scanning laser mode to avoid fluorochrome cross-excitation.

### Tissue Homogenates and Western Blotting

Mouse tissue was homogenized in lysis buffer (50 mM Tris/HCl pH 7.4, 150 mM NaCl, 1% Triton X-100, 1.5 mM MgCl_2_, 10% glycerol, 1 mM PMSF and protease inhibitors) and centrifuged at 15,000 r.p.m for 30 minutes at 4°C. Supernatants containing soluble protein were quantified using the BCA assay (Pierce, Rockford, USA) and the total cell protein extract (50 µg) was mixed with Laemmli sample buffer, boiled at 100°C for 10 minutes and run on 8–12% SDS-PAGE gels. The proteins were transferred to PVDF membranes that were then probed with the corresponding antibodies at the concentrations indicated. Antibody binding was visualized by enhanced chemiluminescence (ECL, Amersham-Pharmacia) and monoclonal antibodies against actin and tubulin were used to normalize for loading.

### Immunohistochemistry

The day of detecting the vaginal plug in female *Prnp^+/0^* mice was considered as embryonic day 0.5 (E0.5) and the day of birth (the night between E19 and E20) was considered postnatal day 0 (P0). Animals were sacrificed on either E16.5 or upon reaching 2 months of age (adults). Three to five animals from at least 3 different litters of different genotypes were processed after genomic identification. Fetuses were removed by caesarean section and all the animals were transcardially perfused with 4% PFA. After perfusion, the brain was removed from the skull and post-fixed in 4% PFA for 12 hours, cryoprotected in 30% sucrose and sectioned on a cryostat at a thickness of 20 (embryos) or 30 µm (adults). The sections were permeabilized with 0.1 M PBS containing 0.2% Triton X-100 and to avoid unwanted cross-reactivity with the immunoreagents they were then blocked with 10% normal goat serum containing anti-mouse or anti-rabbit Fab fragments (1∶50: Jackson ImmunoResearch, West Grove, USA). Subsequently, the sections were incubated overnight at 4°C with the primary antibodies (NG2, Olig2, etc) and the primary antibodies bound to the tissue were detected using the avidin-biotin peroxidase complex (ABC), according to the manufacturer’s instructions (Vector Laboratories), or with Alexa-Fluor 488 and 546 conjugated secondary antibodies (Molecular Probes, Eugene, USA). For the ABC method, immunoreagents were diluted in 0.1 M PBS containing 0.5% Triton X-100, 0.2% gelatin and 5% pre-immune serum. After development with 0.03% diaminobenzidine (DAB) and 0.01% H_2_0_2_, sections were mounted on gelatinized slides, dehydrated in ethanol and coverslipped with Eukitt^TM^ (Merck Chemicals, Darmstadt, Germany). For immunofluorescence, sections were counterstained with DAPI and mounted in Fluoromount^TM^ (Vector Labs). Omission of the primary antibody or its substitution with normal serum in the immunocytochemical controls yielded no immunostaining.

### *In situ* Hybridization

To detect MAG expression, we generated a cRNA probe that recognizes both S-MAG and L-MAG [41]. A 760 bp restriction fragment (base pairs 885–1645, shared by both S- and L-MAG) was obtained by digesting the full-length cDNA with *Eco*RI and *Xho*I and it was cloned into pBlueScript SK^+^. A MAG antisense probe was generated from this plasmid by linearization with *Eco*RI, followed by *in vitro* transcription with T7 RNA polymerase. Conversely, a MAG sense probe was generated by linearization with *Xho*I followed by transcription with T3 RNA polymerase. Both sense and anti-sense riboprobes were labeled with digoxigenin according to the manufacturer’s instructions (Roche Farma, Barcelona, Spain), and *in situ* hybridization was carried out as described previously [41].

### BrdU-pulse Labeling and Immunohistochemistry

For BrdU labeling, 2 month old mice (*Prnp^0/0^ or Prnp^+/+^)* received a daily i.p. pulse of BrdU (50 mg/kg b.w.) on 4 days (see [40] for details). BrdU-injected mice were assigned to 2 equivalent experimental groups and sacrificed 1 (4 + 1) or 15 (4 + 15) days after the last BrdU injection. Mice were perfused with 4% PFA and post-fixed in the same fixative for an additional 2.5 hours at 4°C. After fixation, the brains of the mice were cryoprotected, frozen and microtome sections were obtained (30 µm). Free-floating sections were processed as described previously [42]. Briefly, sections were pre-treated with cold 0.1 N HCl for 15 minutes and 2 N HCl for 20 minutes at 37°C to denature the DNA. After rinsing in 0.1 M PBS, the sections were incubated with a Fab goat anti-mouse IgG (1∶50, Jackson ImmunoResearch) for 2 hours and then with the anti-BrdU antibody. The binding of the primary antibody to the tissue was detected using a biotinylated secondary antibody and the ABC method. Alternative serial sections were stained with cresyl violet or processed for dual immunofluorescence detection of BrdU using Alexa-Fluor 488 and Alexa-Fluor 568 tagged secondary antibodies (Molecular Probes) as indicated [43]. Finally, the sections were mounted in Fluoromount^TM^ (Vector Labs).

### RT-qPCR

Quantitative real time PCR was performed on total RNA extracted from isolated oligodendrocytes with the mirVana’s isolation kit (Ambion) according to the manufacturer’s instructions. Purified RNAs were used to generate the corresponding cDNAs that served as PCR templates for mRNA quantification. The following primers were used for RT-qPCR validation: *Prnp* For: 5′-agtcgttgccaaaatggatca-3′; *Prnp* Rev: 5̀-aaccaacctcaagcatgtgg-3′; O*lig2* For: 5′-ctggtgtctagtcgcccatc-3′; *Olig2* Rev: 5′-gctcagtcatctgcttct-3′; *NG2* For: 5′-agcacgatgactctgagacc-3′; *NG2* Rev: 5′-ggctacgtgaagataggg-3′; *Sox10* For: 5′-cggacgatgacaagttcccc-3′; *Sox10* Rev: 5′-gaggtgagggtactggtcg-3′; *Nkx2.2* For: 5′-ggtggagcgattggataaga-3′; *Nkx2.2* Rev: 5′-tgccatcaaccttttcatca-3′; *CNPase* For: 5′-cagctcaaggagaagaacc-3′; *CNPase* Rev: 5′-ttgtacagtgcagcacacc-3′; *APC* For: 5′-gaagtcagtcggcatctaaagga-3′; *APC* Rev: 5′-tctccaagtactcactcgagg-3′. *Sox17* For: 5′-ctttatggtgtgggccaaag-3′; *Sox17* Rev: 5′-cttctctgccaaggtcaacg-3′; *cdk2* Rev: 5′-cctgcttatcaatgcagaggg-3′; *cdk2* Rev: 5′-tgcgggtcaccatttcagc-3′. PCR amplification and detection was performed on a Roche LightCycler 480 detector, using the 2x Sybr Green Master Mix (Roche) as the reagent according to the manufacturer’s instructions. The reaction protocol involved a denaturation-activation cycle (95°C for 10 minutes), followed by 40 cycles of denaturation-annealing-extension (95°C for 10 minutes, 72°C for 1 minute, 98°C continuous). The mRNA levels were calculated using the LightCycler 480 software and the data was analyzed using the ΔΔ*C*t method. Both experimental and calibration samples were normalized to the relative expression of a housekeeping gene (GAPDH).

## Results

### Impaired Proliferation and Differentiation of Hippocampal Progenitor Cells in PrP^c^ Knockout Mice

**Figure 1:**
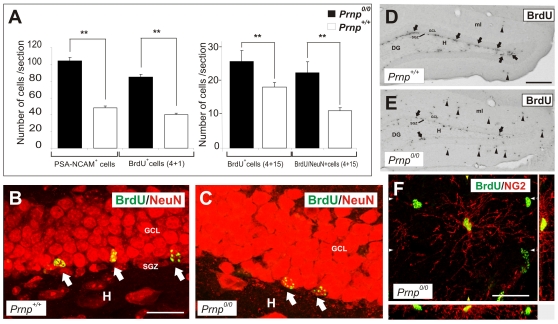
Low SGZ proliferation and neurogenesis in the dentate gyrus of adult *Prnp^0/0^* mice correlate with high numbers of double labeled NG2-BrdU cells in the molecular layer. **A**) Left: Quantification of the number of PSA-NCAM-positive and BrdU-positive cells in dentate gyrus sections from *Prnp^+/+^* and *Prnp^0/0^* mice one day after BrdU-labeling. Right: Quantification of the number of BrdU-positive and BrdU/NeuN-positive cells per section in the dentate gyrus 15 days after administering the last BrdU pulse. Values represent the mean ± standard deviation and the asterisks indicate statistical significance (*P < 0.01*, Student́s *t*-test). **B-C**) Representative photomicrographs showing the suprapyramidal region of the adult dentate gyrus of 2 month old *Prnp^+/+^* (B) and *Prnp^0/0^* (C) mice injected with BrdU 15 days prior to sacrifice. Sections were incubated with antibodies for NeuN and BrdU and the arrows indicate newborn neurons in the dentate gyrus. The fluorochrome used in each case is indicated in the Figure. **D-E**) Representative low power photomicrographs of the dentate gyrus of 2 month old *Prnp^+/+^* (D) and *Prnp^0/0^* (E) mice injected with BrdU 1 day prior to sacrifice. Note the decrease in the number of cells that incorporate BrdU (arrows) in the subgranular zone of *Prnp^0/0^* compared to *Prnp^+/+^* mice. By contrast, numerous BrdU-positive cells (arrowheads) can be seen in the molecular layer of *Prnp^0/0^* than in *Prnp^+/+^* mice. **F**) Example of a double-labeled BrdU/NG2 cells in the molecular layer of *Prnp^0/0^* mice. This image was obtained on an Olympus confocal microscope and processed with Imaris Silicon Graphics software to obtain the orthogonal 3D Z-axis projections. Orthogonal projections are shown on the right (y-axis) and at the bottom (x-axis). Abbreviations: DG: dentate gyrus: GCL: granule cell layer; ML: molecular layer; H: Hilus; SGZ: subgranular zone. Scale bars: B  =  50 µm also applies to C. D  =  200 µm also applies to E; F  =  25 µm.

PrP^c^ expression was previously shown to enhance the proliferation of SVZ-derived stem cells, whilst in its absence the proliferation of hippocampal progenitors in the subgranular zone (SGZ) was impaired [44]. More recently, PrP^c^ was shown to play a critical role in modulating the proliferation of cells in embryonic-derived neurospheres [45]. Thus, we sought to corroborate these results in our backcrossed *Prnp^0/0^* mice. Cell proliferation and neurogenesis in the dentate gyrus of *Prnp^0/0^* and *Prnp^+/+^* mice was monitored by measuring BrdU incorporation into dividing cells 1 or 15 days after administering BrdU (**Fig. 1**). The cell counts in the SGZ and granule cell layer of the dentate gyrus revealed that there was 52.9% reduction in the cells that incorporated BrdU cells in *Prnp^0/0^* mice when compared with the *Prnp^+/+^* animals 1 day after the last BrdU pulse. Moreover, a similar decrease in the number of cells expressing PSA-NCAM (a marker for neuronal lineage in the SGZ [46], [47], [48], [49]) was also detected (**Fig. 1A**). To determine whether the decrease in the numbers of BrdU-positive cells affected neurogenesis in the dentate gyrus, we assessed the incorporation of BrdU in cells that expressed the neuronal marker NeuN 15 days after the last BrdU pulse (**Fig. 1A-C**). While the proportion of cells that incorporated BrdU in *Prnp^0/0^* mice was 32% lower than in *Prnp^+/+^* mice, the Figure rose to 50.7% when considering the cells expressing NeuN that had incorporated BrdU (**Fig. 1A**). Thus, the absence of PrP^c^ diminished the proliferation of neuroprogenitor cells and/or neurogenesis in the adult dentate gyrus. However, a more detailed analysis of the BrdU incorporation revealed a significant increase in cells that incorporated BrdU outside the granular cell layer and in the SGZ of the dentate gyrus of *Prnp^0/0^* mice (**Fig. 1D-E**), and these BrdU-positive cells in the molecular layer also expressed NG2 (**Fig. 1F**). Based on this observation, we sought to determine the possible role of PrP^c^ in oligodendrocyte proliferation and differentiation, both *in vitro* and *in vivo*.

### PrP^c^ is Expressed by OPCs in the Developing and Perinatal Telencephalon

**Figure 2:**
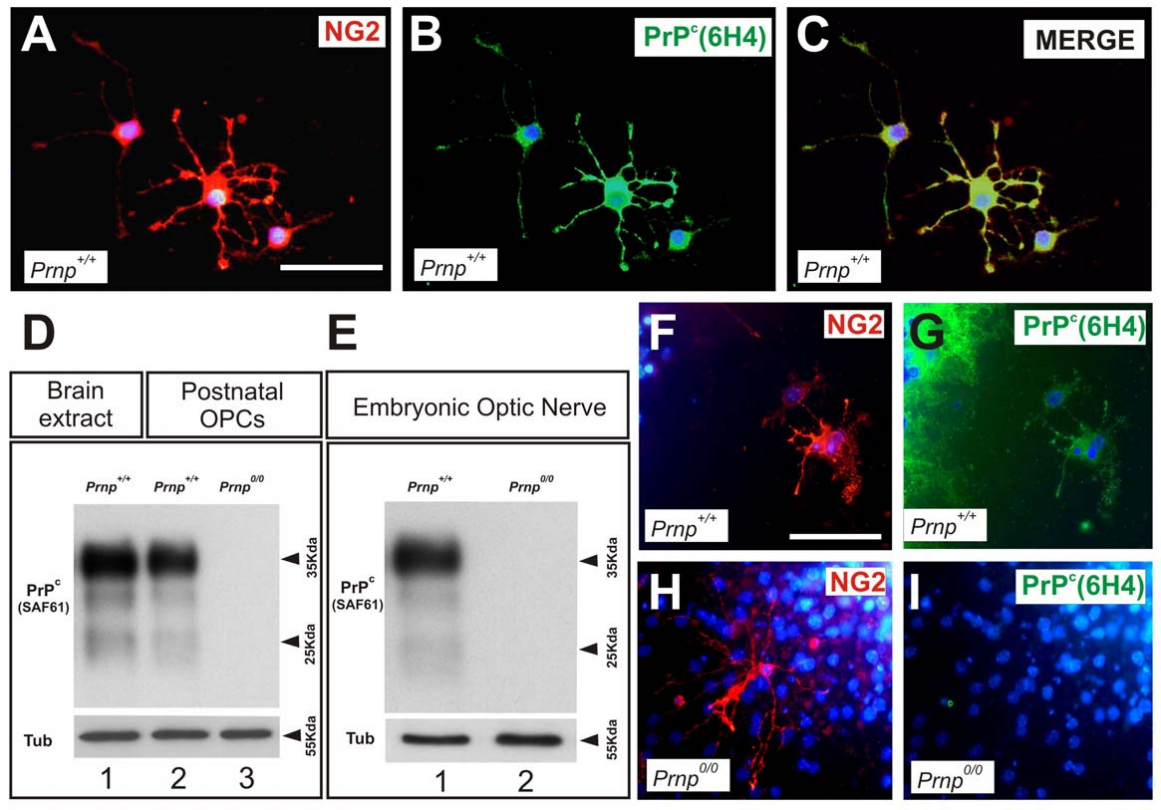
PrP^c^ is expressed in oligodendrocyte precursor cells. **A-C**) High magnification immunofluorescence images of double-labeled (NG2-PrP^c^) cells in primary OPC cultures. **D**) PrP^c^ detection in Western blots of brain extracts from P0 *Prnp* ^+/+^ mice probed with the SAF61 antibody (lane 1) and extracts from primary postnatal oligodendrocyte cultures of *Prnp* ^+/+^ and *Prnp^0/0^* mice (lanes 2 and 3 respectively). **E**) PrP^c^ detection in Western blots of embryonic mice ONs (E16.5) from *Prnp* ^+/+^ (lane 1) and *Prnp^0/0^* mice (lane 2) probed with the SAF61 antibody. No PrP^c^ was detected in ONs from *Prnp^0/0^* mice. **F-I**) Immunofluorescence images of *Prnp^0/0^* (F-G) and *Prnp* ^+/+^ (H-I) SVZ neurospheres after differentiation, demonstrating the ability of both genotypes to differentiate into NG2-positive cells. No PrP^c^ expression was detected in NG2-positive cells in *Prnp^0/0^* neurospheres. Scale bars: A  =  25 µm also applies to B-C; F  =  50 µm also applies to G-I.

In the CNS, PrP^c^ expression has been described in postmitotic neurons and glial cells [44], [50], as well as in isolated oligodendrocytes and myelin [51]. However, there is no strong evidence to date that PrP^c^ is expressed by OPCs and indeed, glial PrP^c^ was not detected in the brain of transgenic mice expressing PrP^c^-eGFP under the *Prnp* promoter [52] or eGFP under the control of the bovine *Prnp* gene promoter [53]. Unfortunately, PrP^c^ is difficult to localize in tissue sections and there are only a few non-commercial antibodies available to detect PrP^c^ (see [52] for technical details). Accordingly, we performed several experiments to define PrP^c^ expression in OPCs. First, we used double immunofluorescence to examine PrP^c^ expression in cultured OPCs that express NG2 from P0 postnatal *Prnp^+/+^* brains (**Fig. 2A-C**). Subsequently, we determined the expression of PrP^c^ in Western blots of protein extracts from cortical OPC obtained from postnatal mice (P0–P2) and from the mouse embryonic ON (**Fig. 2D,E**). The characteristic pattern of PrP^c^ expression was detected in Western blots of *Prnp^+/+^* OPC extracts [54] but it was absent from extracts from *Prnp^0/0^* mice. Moreover, we determined PrP^c^ expression in NG2-positive cells differentiated from SVZ neurospheres and maintained for 20 weeks as floating aggregates in the presence of EGF and FGF-2 before differentiating for 7 days without growth factors (**Fig. 2F-I**). As expected, NG2-positive cells from *Prnp^0/0^*-derived SVZ neurospheres exhibited no PrP^c^ staining (**Fig. 2H-I**).

### The Proliferation of OPCs is Stronger in ON Explants from *Prnp^0/0^* Embryos

**Figure 3:**
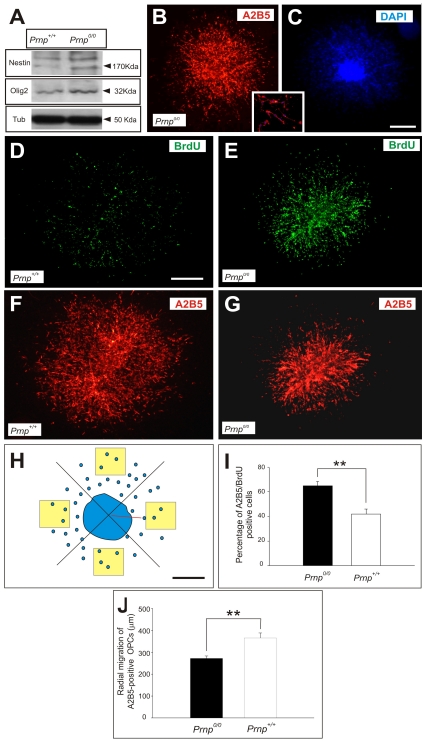
The absence PrP^c^ increases OPC proliferation in cultured embryonic ONs. **A**) Western blots of embryonic ONs (E16.5) from *Prnp^0/0^* and *Prnp* ^+/+^ mice probed for Nestin and Olig2. Expression of both proteins was increased in *Prnp^0/0^* versus *Prnp* ^+/+^ ONs. **B-C**) Representative immunofluorescence images of a 2 DIV ON explant culture from *Prnp* ^+/+^ mice in three dimensional collagen matrices after staining with anti-AB25 and counterstaining with DAPI. Cells that migrated radially were identified as OPCs by the expression of the A2B5 epitope (see also lower box). **D-G**) Immunofluorescence photomicrographs of 2 DIV ON explant cultures from *Prnp* (D-E) and *Prnp^0/0^* (F-G) mice in three dimensional matrices after double staining with anti-BrdU and anti-A2B5. **H**) Scheme illustrating the method to quantify A2B5 and BrdU staining in explant cultures. Yellow boxes to quantify each quadrant are shown. **I**) Histogram showing relative percentage of A2B5 and BrdU-positive cells in *Prnp^0/0^* and *Prnp^+/+^* mice. The absence of PrP^c^ significantly increased the number of BrdU-labeled OPCs. **J**) Quantification of the maximal distance migrated by OPCs in ON explants. *Prnp* ^+/+^ OPCs migrated significantly further than their *Prnp^0/0^* counterparts. Values in I and J represent the mean ± standard deviation, and the asterisks indicate statistical significance (*P* < 0.01, Student́s *t*-test). Scale bars: in B and D  =  200 µm also apply to A, and E-G, respectively.

We next analyzed OPC proliferation in embryonic ON explants from *Prnp^0/0^* and *Prnp^+/+^* embryos (**Fig. 3**). At the developmental stages studied, most of the cells populating the ON were proliferating (Nestin-positive) oligodendrocyte precursors (Olig2-positive). To study these two populations quantitatively, we analyzed ON protein extracts in Western blots, which revealed that more Nestin and Olig2 were found in the *Prnp^0/0^* ON than in the corresponding *Prnp^+/+^* extracts (**Fig. 3A**). When ON explants from the two genotypes were cultured on collagen matrices and incubated with BrdU (see methods), no astrocytes migrated from the explants and only OPCs identified as A2B5-positive cells migrate radially from the explants [33], [34], [55] (**Fig. 3B,C**). Indeed, more cells incorporated BrdU in the *Prnp^0/0^* explants than in those from *Prnp^+/+^* mice (**Fig. 3D,E**), and after A2B5 immunostaining, *Prnp^+/+^* OPCs clearly migrated further from the explants (**Fig. 3F,G**). To corroborate these observations, we counted the number of double-labeled (A2B5-BrdU) cells in four quadrants (200 µm^2^, yellow squares in **Fig. 3H,I**). Cell counts in randomly selected quadrants of *Prnp^0/0^* cultures revealed a 23% increase in the number of A2B5 expressing cells that had incorporated BrdU than in the *Prnp^+/+^* cultures (**Fig. 3I**). By contrast, there was a 31% decrease in the distance migrated by *Prnp^0/0^* derived OPCs when compared with *Prnp^+/+^* OPCs (**Fig. 3J**).These data revealed a clear correlation between the proliferation and migratory potential in OPCs from *Prnp^0/0^* ON explants, with higher proliferation associated with lower migration. Indeed, *Prnp^+/+^* OPCs displayed weaker proliferative activity but increased migration. These findings suggest that in three dimensional hydrogels, PrP^c^ decreased the proliferation and increased the migratory properties of ON-derived oligodendrocytes.

### The Absence of PrP^c^ in Isolated Cortical OPCs Augments Proliferation and Delays Differentiation

**Figure 4:**
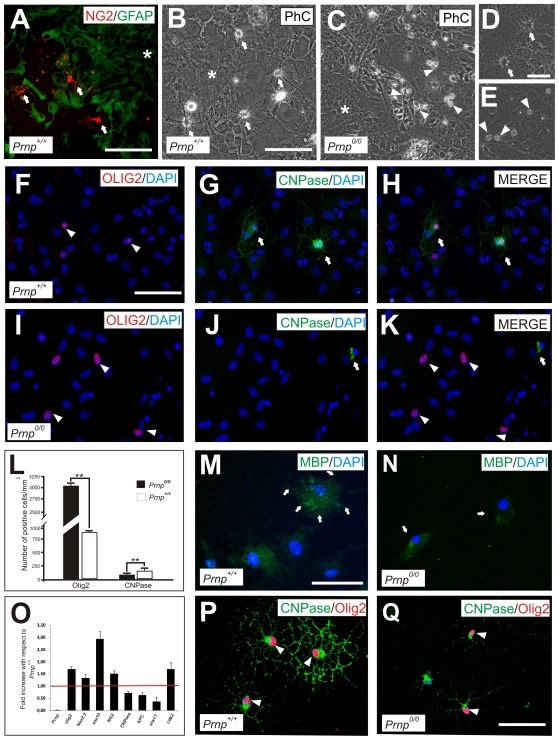
Cultured *Prnp^0/0^* oligodendrocytes remain in an undifferentiated state for longer than the *Prnp* ^+/+^ controls. **A**) Low power photomicrograph of a primary *Prnp* ^+/+^ mixed culture showing NG2-positive oligodendrocytes (arrows) grown and differentiated over a GFAP-positive astrocyte monolayer. **B-C**) Phase contrast (PhC) images of a *Prnp* ^+/+^ (B) and *Prnp^0/0^* (C) primary oligodendrocyte mixed cultures showing the morphology after 3 days in SFM. Numerous ramified oligodendrocytes (arrows in B) can be seen in *Prnp^0/0^*-derived cultures, in contrast to the bipolar oligodendrocyte morphologies (arrowheads in C) seen over the astrocyte monolayer (asterisk in B and C). **D-E**) High magnification images of B and C showing the greater ramification (arrows) of *Prnp^+/+^* versus *Prnp^0/0^* (arrowheads) oligodendrocytes. **F-K**) Immunofluorescence images of Olig2 (F,I) and CNPase (G,J) expression in mixed cultures (5 days in SFM) derived from *Prnp* ^+/+^ and *Prnp^0/0^* mice. Cultures were counterstained with DAPI and the arrows indicate the CNPase-Olig2-positive cells in both cultures. Note the absence of double labeled (CNPase/Olig2) cells in *Prnp^0/0^* derived cultures. **L**) Histogram illustrating the mean number of Olig2 and CNPase cells per mm^2^ in cultures of both genotypes. Values represent mean ± standard deviation and the asterisks indicate statistical significance (*P* < 0.01 Student́s *t*-test). **M-N**) MBP labeling in *Prnp^+/+^* (M) and *Prnp^0/0^* (N) in mixed cultures after 5 days in SFM. **O**) Histogram showing RT-qPCR analysis of RNA samples extracted from *Prnp^+/+^* and *Prnp^0/0^* purified oligodendrocytes from mixed cultures after 5DIV in SFM (see methods for details). Data represent the mean induction of three independent experiments where GAPDH was used as the reference gene. **P-Q**) Immunofluorescence images of double labeled CNPase-Olig2-positive cells in isolated OPCs cultures (5 days in SFM, without astrocytes) derived from *Prnp^+/+^* (P) and *Prnp^0/0^* (Q) mice. Cultures were counterstained. In the *Prnp^0/0^* cultures there were less oligodendrocytes (Olig2-positive arrowheads) expressing CNPase with reduced ramifications (Q) compared to wild-type oligodendrocytes (P). Scale bar: A  =  50 µm; B  =  50 µm also applies to C; D  =  20 µm also applies to E; F  =  50 µm also applies to G-K. M  =  50 µm also applies to N. P  =  40 µm also applies to Q.

OPC primary cultures were established from *Prnp^0/0^* and *Prnp* ^+/+^ mouse pups (P0-P2) as described previously [36], and the presence of OPCs (identified as NG2-positive cells) growing over an astrocyte monolayer was determined by dual immunohistochemistry (**Fig. 4A**). After 3 DIV, there were many ramified oligodendrocytes in cultures derived from *Prnp^+/+^* mice, which were clearly visible by phase contrast microscopy (**Fig. 4B,D**). By contrast, *Prnp^0/0^* cultures contained unramified bipolar cells (**Fig. 4C,E**). To define the putative delay in OPC maturation in these cultures, we performed dual immunohistochemistry 5 DIV after the addition of SFM medium to examine markers of different stages of oligodendrocyte maturation: Olig2, CNPase (**Fig. 4F-K**) and MBP (**Fig. 4M,N**). *Prnp^0/0^* cultures contained fewer double-labeled (CNPase-Olig2) oligodendrocytes than *Prnp^+/+^* cultures, in which ramified double-labeled oligodendrocytes were frequently observed (**Fig. 4F-L**), and there was a 2.6-fold increase in CNPase-positive cells (123 ± 13 cells/mm^2^) in the *Prnp^+/+^* cultures with respect to the *Prnp^0/0^* cultures (47.6 ± 11 cells/mm^2^). Conversely, the number of Olig2 cells was 3.72-fold greater in primary *Prnp^0/0^* versus *Prnp^+/+^* cultures (3,033 ± 136 *vs* 816 ± 113 cells/mm^2^, respectively: **Fig. 4L**).

When we studied markers of further differentiation, such as MBP, *Prnp^0/0^* derived oligodendrocyte cultures exhibited milder MBP staining than *Prnp^+/+^* cultures in which several multipolar cells displayed considerable MBP staining (**Fig. 4M,N**). Taken together, these results suggest that there is a delay in oligodendrocyte differentiation in *Prnp^0/0^* cultures. To corroborate these findings, we assessed the expression of oligodendrocyte markers in mRNA extracted from oligodendrocytes in mixed cultures after 5DIV in SFM and we found that *Olig2*, *Sox10* and *Nkx2.2* were mildly up-regulated in oligodendrocytes derived from *Prnp^0/0^* cultures (**Fig. 4O**). However, when markers of different stages of oligodendrocyte differentiation were analyzed there was an up-regulation of *NG2* in *Prnp^0/0^* cultures while other markers of maturation were down-regulated, such as *Sox17*, *CNPase* and *APC* (**Fig. 4O**). These data suggest that the absence of PrP^c^ delays oligodendrocyte differentiation *in vitro*. Finally, we analyzed the differential expression cyclin-dependent kinase 2 (cdk2), a marker of the cell cycle that controls the progression of oligodendrocytes through the cell cycle and their differentiation [56], [57]. Cdk2 expression was significantly up-regulated in oligodendrocytes derived from *Prnp^0/0^* cultures, consistent with the immature oligodendrocyte phenotype observed in *Prnp^0/0^* cultures.

Given that we were working with mixed cultures and since astrocytes also express PrP^c^ (**Fig. S1**), we examined whether astrocyte conditioned media from *Prnp^+/+^* astrocytes influenced OPC differentiation in *Prnp^0/0^* cultures and *vice versa*. Results showed a lower percentage of CNPase/Olig2 cells in *Prnp^0/0^* OPC cultures than in *Prnp^+/+^* irrespective of the amount of the added astrocyte conditioned media (**Fig. S1**). To further rule out the potential influence of astrocytes on OPC differentiation, isolated OPCs from *Prnp^0/0^* and *Prnp^+/+^* mice were maintained in SFM medium (see methods). After 5 DIV, OPCs derived from *Prnp^+/+^* mice again displayed more CNPase-positive ramifications than those from *Prnp^0/0^* mice (**Fig. 4P,Q**). Moreover, fewer oligodendrocytes were double-labeled (CNPase/Olig2) in *Prnp^0/0^* cultures compared to *Prnp^+/+^* ones (**Fig. S2**). To corroborate these findings, we analyzed the mRNA isolated from these oligodendrocytes at 0 and 5 DIV in SFM. We observed similar tendencies in these cultures to the mixed cultures and again, oligodendrocyte markers were up-regulated in knockout mice (*Olig2* and *NG2*) while differentiation markers were downregulated, such as *Sox17* (**Fig. S2**). Together, these data confirm that the differences in the oligodendrocyte differentiation in absence of PrP^C^ are not mediated by astroglial cells.

### Olig2*-* and NG2-positive Cells are More Abundant in *Prnp^0/0^* than in *Prnp^+/+^* Mice at Perinatal and Adult Stages

**Figure 5:**
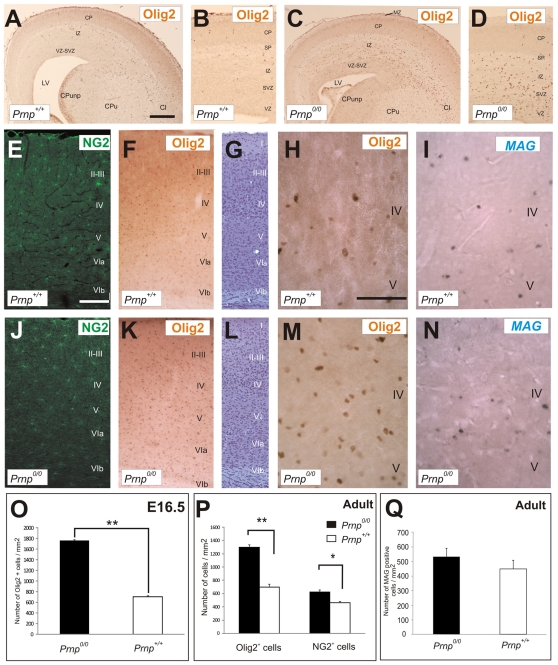
PrP^c^ expression modulates the number of oligodendrocytes in the perinatal and adult neocortex. **A-D**) Low power photomicrographs showing Olig2 expression in coronal sections of *Prnp* ^+/+^ and *Prnp^0/0^* embryonic (E16.5) brains. **B,D**) High magnification photomicrographs of A and C. **E-N**) Low power photomicrographs showing a coronal brain section immunostained for NG2 (E and J), Olig2 (F, H, K and M) and MAG. *In situ* hybridization (I and N) of the parietal cortex of adult *Prnp* ^+/+^ (E-I) and *Prnp^0/0^* mice (J-N). G and L are adjacent Nissl stained sections to E and J. Note the increase in the number of NG2- and Olig2-positive cells in the *Prnp^0/0^* neocortex compared to that of the *Prnp^+/+^* mice. **O**) Histogram showing the number of Olig2-positive cells in the embryonic neocortex of *Prnp^0/0^* and *Prnp* ^+/+^ mice. **P**) Histogram showing the number of Olig2- and NG2-positive cells in the adult neocortex of *Prnp^0/0^* and *Prnp* ^+/+^ mice. The presence of Olig2- and NG2-positive cells increased significantly in the knockout mice when compared with their *Prnp^+/+^* counterparts. **Q**) Quantification of the number of MAG-positive cells in the adult cortex of *Prnp^0/0^* and *Prnp* ^+/+^ mice, revealing no significant differences between these genotypes. Values represent the mean ± standard deviation and the asterisks indicate statistical significance (*P* < 0.01, Student́s *t-* test). Scale bars: A  =  500 µm also applies to C; B  =  200 µm also applies to D. E  =  300 µm also applies to F,G and J-L; H  =  100 µm also applies to I and M-N.

**Figure 6:**
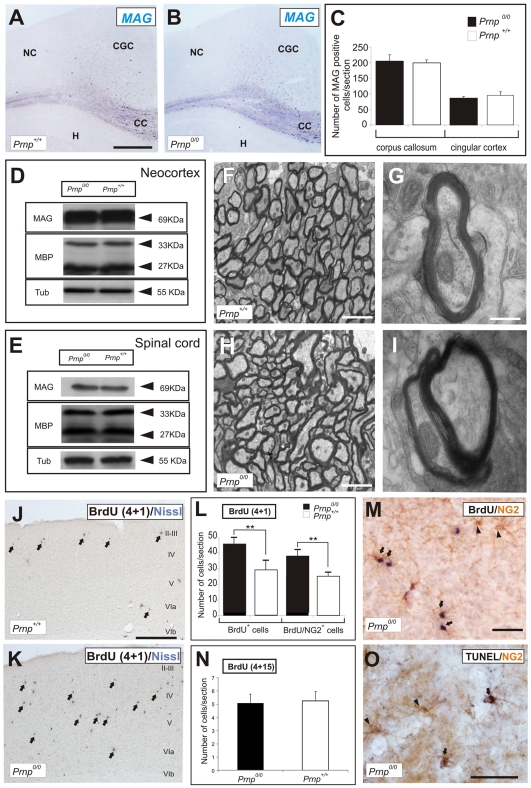
Adult *Prnp^0/0^* mice exhibit normal myelination in the CNS. **A-B**) *In situ* hybridization of *MAG* in coronal sections from the brains of adult *Prnp^+/+^* and *Prnp^0/0^* mice. **C**) Quantification of MAG-positive cells revealed no differences between genotypes in the corpus callosum or cingular cortex. Values represent the mean ± standard deviation and they were analyzed using the Student’s *t*-test. **D-E**) Western blots of brain extracts from *Prnp^+/+^* and *Prnp^0/0^* mice showing no differences in the expression of the MAG and MBP myelin proteins between genotypes in neocortex (D) and spinal cord (E). **F, H**) Lower magnification electron microscopy photomicrograph of *Prnp* ^+/+^ and *Prnp^0/0^* mice. **G, I**) Higher magnification of F and H, respectively, showing no gross ultrastructural differences in the myelin sheaths of the corpus callosum. **J-K**) Representative photomicrographs of the parietal cortex of *Prnp^+/+^* (J) and *Prnp^0/0^* (K) mice immunostained for BrdU 1 day after the last BrdU pulse. Slides were lightly counterstained with Nissl. Numerous BrdU-positive cells can be seen in *Prnp^0/0^* compared to wild-type (arrows in J and K) **L**) Quantification of BrdU incorporation and the double-labeled BrdU-NG2-positive cells 1 day after the last BrdU pulse. Both populations increased in the parietal cortex of *Prnp^0/0^* mice when compared to *Prnp^+/+^* mice. **M**) Photomicrograph of double-labeled BrdU-NG2 cells (arrows) in the parietal cortex of *Prnp^0/0^* mice. Arrowheads indicate non-BrdU NG2-positive cells. **N**) Quantification of BrdU-positive cells 15 days after the BrdU pulse (4 + 15 protocol). Values in (L) and (N) represent the mean ± standard deviation and the asterisks indicate statistical significance (*P* < 0.01, Student *t*-test). **O**) Photomicrograph showing double-labeled NG2/TUNEL cells (arrows) in the *Prnp^0/0^* parietal cortex, in which the arrowheads indicate non-NG2-TUNEL-positive cells. Abbreviations: DG: dentate gyrus: GCL: granule cell layer; ML: molecular layer; H: Hilus; SGZ: subgranular zone. CC: corpus callosum; CGC: cingulate cortex; NC: neocortex. H: hippocampus; Scale bars: A  =  300 µm also applies to B; F: 2 µm also applies to H; G  =  0.5 µm also applies to I; J and K  =  100 µm. M  =  50 µm and O  =  25 µm.

When we next investigated the presence of OPCs during perinatal development, we found that Olig2-immunoreactive cells that migrated tangentially from subpalial regions were more abundant (2.5-fold) in the neocortex of *Prnp^0/0^* than in *Prnp^+/+^* embryos (E16.5: **Fig. 5A-D,O**). Indeed, there was an increase in cells expressing Olig2 and NG2 (**Fig. 5P**) in sections of the parietal cortex of adult *Prnp^0/0^* versus *Prnp^+/+^* mice (**Fig. 5E,F,J,K,H,M**). Moreover, these results correlated with the 1.44- and 1.35-fold increase in *Olig2* and *NG2* mRNA in this region, as determined by RT-qPCR (**Fig. S3**). Nissl counterstaining revealed no differences between *Prnp^+/+^* and *Prnp^0/0^* mice in the organization of the distinct layers in the adult cortex (**Fig. 5G,L**). Moreover, *in situ* hybridization to study the expression of the myelin-associated glycoprotein (MAG), identifying mature myelinating oligodendrocytes, revealed no significant changes in the neocortex (**Fig. 5I,N,Q**), cingular cortex or the white matter of *Prnp^0/0^* mice (**Fig. 6A-C**). Accordingly, myelination did not appear to be affected by the absence of *Prnp*. Indeed, no significant differences were detected between the two genotypes in terms of the amount of total MAG or myelin basic protein (MBP) in adult cortical (**Fig. 6D**) and spinal cord extracts (**Fig. 6E**). In addition, electron microscopy revealed no alterations to the myelin sheaths in the cortical white matter due to the loss of *Prnp* (**Fig. 6F-I**). In conclusion, our results indicate an increase in the number of NG2- and Olig2-positive cells in the neocortex of *Prnp^0/0^* mice, which were not correlated with defective myelination. These results are consistent with the normal myelin content and structure described previously in the CNS of *Prnp^0/0^* mice (see Introduction).

**Figure 7:**
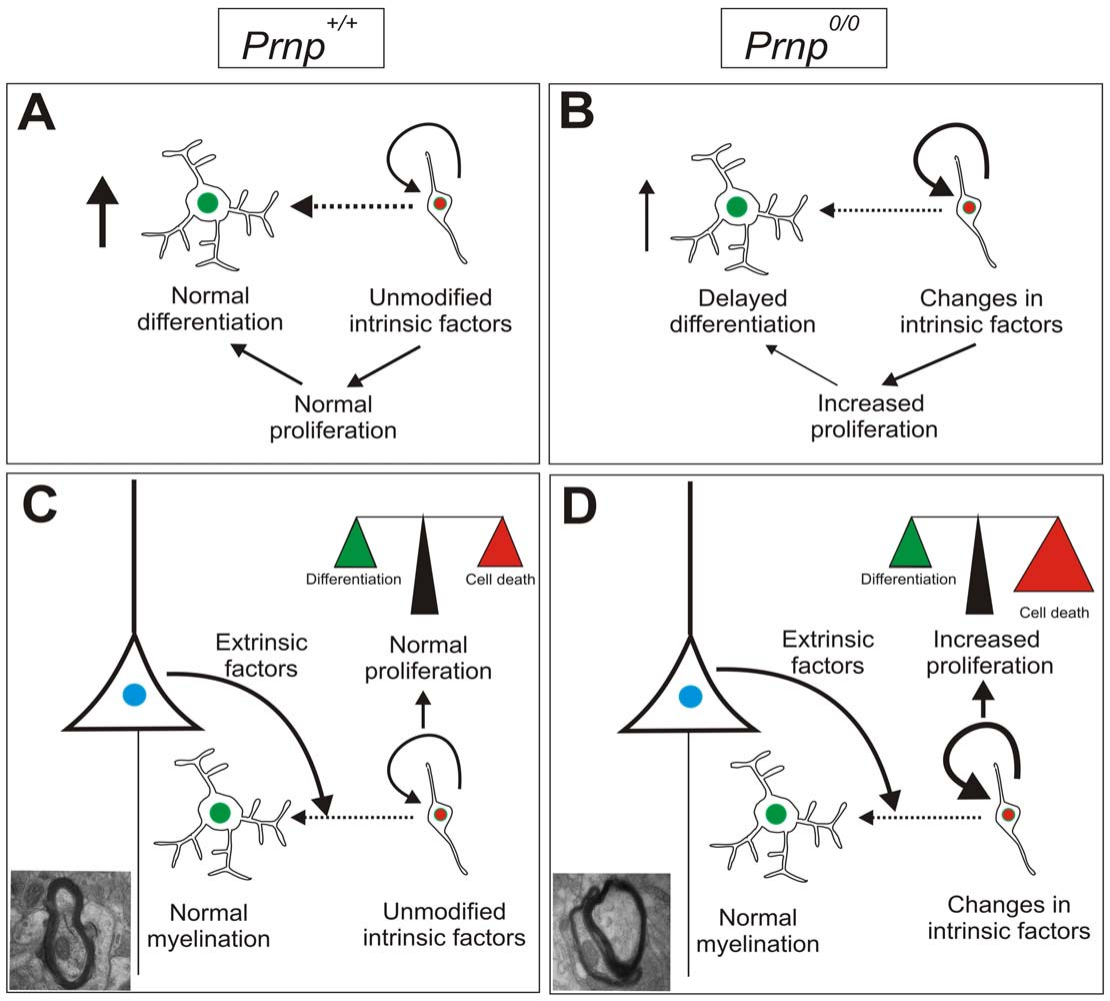
A putative scheme of PrP^c^ involvement in oligodendrocyte proliferation and maturation. **A-B**)**.** Scheme summarizing the main *in vitro* findings of the present study. The absence of PrP^c^ in cultured OPCs from the ON and in the isolated cortical OPCs prolonged the proliferative stage in these precursors, and the modification of intrinsic factors modulating cell proliferation and oligodendrocyte differentiation (e.g., *Sox17, cdk2)* may delay their normal maturation. **C-D**) No differences in CNS myelination were observed between *Prnp^0/0^* and *Prnp^+/+^* mice, although increased proliferation was observed in adult NG2 cells in *Prnp^0/0^* mice. The putative changes in intrinsic factors are overcome by extrinsic (neuronal and astroglial) factors to establish normal myelination. These extrinsic signals (*e.g.,* PDGF-AA, FGF-2, IGF-1, NT-3 or CNTF) may help to control the proper timing of OPC differentiation, ensuring adequate myelination and its maintenance. Surplus NG2 cells are eliminated by cell death in this balanced system.

### The Number of NG2-positive Cycling Cells Increased in the Neocortex of Adult *Prnp^0/0^* Mice

We extended our studies on BrdU accumulation and examined the effects of pulse labeling in adult mice. One day after the last BrdU pulse, more cells had incorporated BrdU in all the cortical areas and subcortical regions of adult *Prnp^0/0^* mice than in *Prnp^+/+^* mice. We also observed more cells that incorporated BrdU in all layers of the parietal cortex in *Prnp^0/0^* mice (43.85 ± 4.4 as opposed to 29.02 ± 2.1 cells per 1.5 mm^2^ in *Prnp^+/+^* mice: **Fig. 6J,K**). Most of the cycling cells in the neural parenchyma that incorporated BrdU expressed NG2 (35.88 ± 4.1 and 24.9 ± 1.7 in *Prnp^0/0^* and *Prnp^+/+^* mice, respectively: **Fig. 6L**), and these cells (NG2-BrdU) were often arranged in small clusters or pairs (**Fig. 6M**). However, 15 days after the last BrdU pulse (4 + 15 days after the first pulse), there were no significant differences between *Prnp^0/0^* and *Prnp* ^+/+^ mice in terms of the number of cells that had incorporated BrdU (5.1 ± 0.7 *vs*. 5.6 ± 0.8, respectively: **Fig. 6N**). These data suggest that the initial overproduction of BrdU labeled OPCs was subsequently attenuated by cell death. Indeed, when we assessed the apoptotic cell death among surplus NG2 cells using the TUNEL assay, some double-labeled NG2-TUNEL-positive cells were observed in the *Prnp^0/0^* parietal cortex (**Fig. 6O**) but not in the sections of *Prnp* ^+/+^ mice. After quantification, we observed a slight increase in the number of NG2/TUNEL cells in *Prnp^0/0^* mice when compared to the wild-type animals (**Fig. S4**). Although a direct correlation cannot be established since *Prnp^0/0^* cells are very sensitive to serum withdrawal, a similar increase in the number of apoptotic cells was also observed in the *Prnp^0/0^* cultures *in vitro* (**Fig. S4**).

## Discussion

Despite numerous studies into the role of PrP^c^ and its pathogenic isoform in transmissible spongiform encephalopathies (TSEs), its function in healthy mammalian neural and non-neuronal cells remains unclear. Opposing effects of PrP^c^ expression on cell proliferation have been reported and there is considerable variation in the relative expression of this protein in different proliferating cell types. For example, murine and bovine PrP^c^ is expressed strongly in proliferating cells such as hematopoietic stem cells, spermatogonia and lymphocytes [58], [59], [60], [61], whereas it is absent in other proliferating cells such as neural stem cells in the SVZ [44] or gut [27]. Taken together, it appears that PrP^c^ expression may modulate the cell cycle and proliferation in a cell specific manner, as demonstrated here. Indeed, we showed that PrP^c^ plays distinct roles in the proliferation of SGZ cells and OPCs. We examined the effect of *Prnp* gene expression on oligodendrocyte differentiation in detail and we demonstrated that the absence of PrP^c^ in OPCs increases their rate of proliferation, strongly implicating PrP^c^ expression in oligodendrocyte differentiation. This finding is consistent with previous reports of higher proliferative rates and shorter duplication times in neural cell lines derived from *Prnp^0/0^* mice when compared with those expressing PrP^c^ [62].

Our results demonstrate that OPCs from the embryonic ON proliferate more in the absence of PrP^c^, both in perinatal stages and in the adult neuronal parenchyma. However, these effects are cell-specific and cannot be generalized to other cell types. For example, treatment with specific antibodies against PrP^c^ inhibits the proliferation of human T lymphocytes after they are activated [63], while re-expression of the *Prnp* gene in splenocytes derived from *Prnp^0/0^* mice restores their proliferative potential [64]. Similar findings were described in gastric cell lines [65] and epithelial cells [66], and human embryonic stem cells (hESCs) proliferation augments when they are incubated with recombinant PrP^c^ due to the down-regulation of PrP^c^ [67]. Whether these effects are mediated by the down-regulation of PrP^c^ *per se* or through specific PrP^c^-mediated intracellular signaling remains unclear, as does the question regarding whether PrP^c^ acts directly (through its aggregation in lipid rafts) or indirectly (by binding to cell receptors such as the laminin receptor (65) or to extracellular factors like STI1 [45]). The identification of PrP^c^ targets that influence the cell cycle in specific cell types may shed light on these issues. Interestingly, we found stronger cdk2 expression in cultured OPCs derived from *Prnp^0/0^* mice. Cdk2 participates in the G1-S transition, and it is crucial for OPC proliferation and differentiation [56], [57]. In fact, contrasting patterns of cdk2 expression have been described in proliferation and differentiation states [56]. However, we cannot rule out the possibility that extrinsic factors modulate OPC proliferation and the increased expression of stress markers in the *Prnp^0/0^* mouse brain [38]. This increase in stress may enhance the proliferation of OPCs in mutant mice as described for other neural cells (*e.g.,* microglia).

To the best of our knowledge, this is the first report of oligodendrocyte differentiation *in vitro* in the absence of PrP^c^. The absence of *Prnp* was correlated with an increase in the expression of OPC markers (*Olig2*, *Sox10*, etc) and the down-regulation of *APC, CNPase* and *Sox17,* particularly in cultured OPCs. *Sox17* controls the transition from the proliferative to the myelinating stage during oligodendrocyte development [68], and it modulates the expression of myelin-associated genes like MBP [68]. Indeed, we propose that the down-regulation of *Sox17*, together with that of other regulators of cell fate, such as cdk2 (see above), is involved in the delayed differentiation of *Prnp^0/0^*-derived OPCs *in vitro*, although further studies will be necessary to confirm this hypothesis. However, in a distinct model it was demonstrated that the undifferentiated state was protracted in *Prnp-*deficient stem cells after serum withdrawal, as determined by the expression of Nestin as opposed to MAP2 protein [44]. Delayed maturation of cerebellar granule cells was recently reported in *Prnp^0/0^* mice, mainly due to the protracted proliferation of granule cells during the postnatal period [69], although normal motor behavior was recovered around postnatal day 50. Similarly, a recent study showed that cells PrP^C^ expression contributes to neuritogenesis [70].

Oligodendrocyte maturation is a complex process and compensatory mechanisms may be active during development and in adulthood. Such mechanisms could compensate for the absence of PrP^c^, resulting in normal differentiation to mature myelinating oligodendrocytes of a proportion of OPCs. This tentative hypothesis may explain why despite the overproduction of OPCs, oligodendrocytes that manage to ensheath the axon survive in *Prnp^0/0^* mice while those that fail degenerate. Oligodendrocyte survival is regulated by their interaction with axons, which serves to ensure that the correct number of oligodendrocytes is matched to the surface of axons requiring ensheathment ([71], see also [72] for discussion). Moreover, natural elimination of overproduced OPCs by programmed cell death targeting OPCs that do not fully mature has also been described [73]. *In vivo,* the direct contact of OPCs with neurons and axonal tracts is maintained in a dynamic manner, and their differentiation is dependent on intrinsic as well as extrinsic cues provided by neurons (see [14], [72], [74] for recent reviews). Thus, although large OPC populations are observed in PrP^c^-deficient mice, it is feasible that similar numbers of OPCs mature in *Prnp^0/0^* and *Prnp^+/+^* mice due to these neural factors and axonal interactions (**Fig. 7**), neither of which influence this process *in vitro*. Accordingly, overproduction of OPCs that do not mature into myelinating oligodendrocytes through the appropriate neuronal-glia interactions may disappear due to cell death (**Fig. 7**). Future studies will investigate the relationship between PrP^c^ and the expression of neuronal factors that modulate OPC differentiation (*e.g.,* PDGF-AA, neuregulins, Notch, etc.), which may be of special interest when considering oligodendrocyte maturation in demyelinating diseases and other myelopathies. In fact, the absence of PrP^c^ accelerates the onset of experimental autoimmune encephalomyelitis (EAE) and exacerbates its devastating effects [75], [76]. Although warrant for further studies, the present data suggest that in absence of PrP^C^, OPCs might remain in a more protracted undifferentiated state that may negatively affect the normal course of differentiation to myelinating oligodendrocytes in the EAE model.

## Supporting Information

Figure S1 **Astrocytes do not affect oligodendrocyte differentitation** ***in vitro*** **.** **A**) Western blots of protein extracts from astrocyte cultures from *Prnp* ^+/+^ mice and total brain, probed with GFAP and PrP^c^ antibodies. **B**) Histogram showing the percentage of CNPase/Olig2-positive cells in cultures of *Prnp^0/0^* and *Prnp* ^+/+^ mice in the presence of different amounts of conditioned media collected from astrocyte cultures of the opposite genotype (0, 25, 50 and 75% of conditioned media in SFM medium). Values represent the mean ± standard deviation and the asterisks indicate statistical significance (P < 0.01, Student t-test). In wild-type cultures there were no differences in differentiation in the presence of *Prnp^0/0^* astrocyte conditioned media, although there were significantly more CNPase/Olig2-positive cells than in knockout cultures. In *Prnp^0/0^* OPCs cultures there were no differences in differentiation in the presence of conditioned media from wild-type astrocyte cultures.
(TIF)

Figure S2 ***Prnp^0/0^*** **oligodendrocytes were less differentiated than the wild type oligodendrocytes in** ***vitro*** **.** **A**) Histogram showed the quantification of the CNPase/Olig2 double labeled cells in cultures of isolated OPCs from *Prnp^0/0^* and *Prnp* ^+/+^ mice. Note that in *Prnp^0/0^* cultures the proportion of mature oligodendrocytes was lower. Values represent the mean ± standard deviation, and the asterisks indicate statistical significance (P < 0.01, Student́s t-test). **B**) Histogram showing RT-qPCR analysis of RNA samples extracted from *Prnp* ^+/+^ and *Prnp^0/0^* purified oligodendrocytes after 0 and 5 DIV in SFM and without astrocytes. The data represent the induction of three independent experiments, with GAPDH used as the reference gene.
(TIF)

Figure S3 ***Prnp^0/0^*** **mice have increased levels of** ***Olig2*** **and** ***NG2*** **mRNA than** ***Prnp*** **^+/+^ mice.** Histogram showing RT-qPCR analysis of RNA samples extracted from the adult *Prnp* ^+/+^ and *Prnp^0/0^* mouse cortex. Data represent the mean induction of three independent experiments in which GAPDH was used as the reference gene.
(TIF)

Figure S4 **PrP^c^ absence derived in OPCs less survival** ***in vivo*** **and** ***in vitro*** **.** **A**) Histogram showed the number of NG2/TUNEL-positive cells in the neocortex of adult *Prnp* ^+/+^ and *Prnp^0/0^* mice. **B**) Histogram showed the number of double labeled Olig2/TUNEL cells in isolated oligodendrocytes derived from *Prnp* ^+/+^ and *Prnp^0/0^* cultures. In both cases there were more apoptotic oligodendrocytes in the absence of PrP^c^. Values in A and B represent the mean ± standard deviation, and the asterisks indicate statistical significance (P < 0.01, Student́s t-test).
(TIF)
