# Supplementary figures and images for: Role of the Cellular Prion Protein in Oligodendrocyte Precursor Cell Proliferation and Differentiation in the Developing and Adult Mouse CNS
Source: PLoS One. 2012 Apr 18;7(4):e33872. doi: 10.1371/journal.pone.0033872 (PMC3329524; doi:10.1371/journal.pone.0033872)

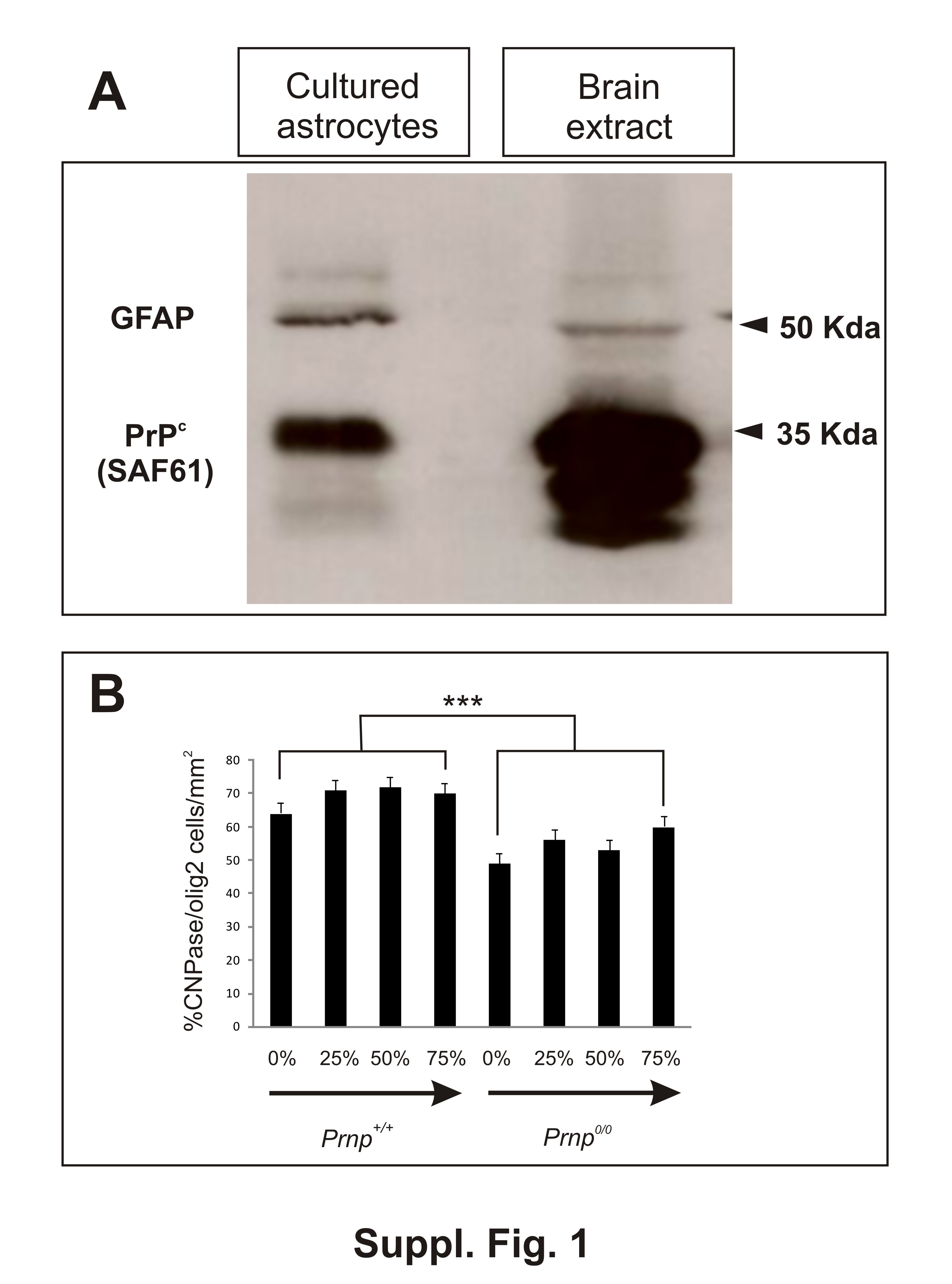

Supplement: Figure S1 — Astrocytes do not affect oligodendrocyte differentitation in vitro . A) Western blots of protein extracts from astrocyte cultures from Prnp +/+ mice and total brain, probed with GFAP and PrPc antibodies. B) Histogram showing the percentage of CNPase/Olig2-positive cells in cultures of Prnp0/0 and Prnp +/+ mice in the presence of different amounts of conditioned media collected from astrocyte cultures of the opposite genotype (0, 25, 50 and 75% of conditioned media in SFM medium). Values represent the mean ± standard deviation and the asterisks indicate statistical significance (P < 0.01, Student t-test). In wild-type cultures there were no differences in differentiation in the presence of Prnp0/0 astrocyte conditioned media, although there were significantly more CNPase/Olig2-positive cells than in knockout cultures. In Prnp0/0 OPCs cultures there were no differences in differentiation in the presence of conditioned media from wild-type astrocyte cultures. (TIF) [file pone.0033872.s001.tif]

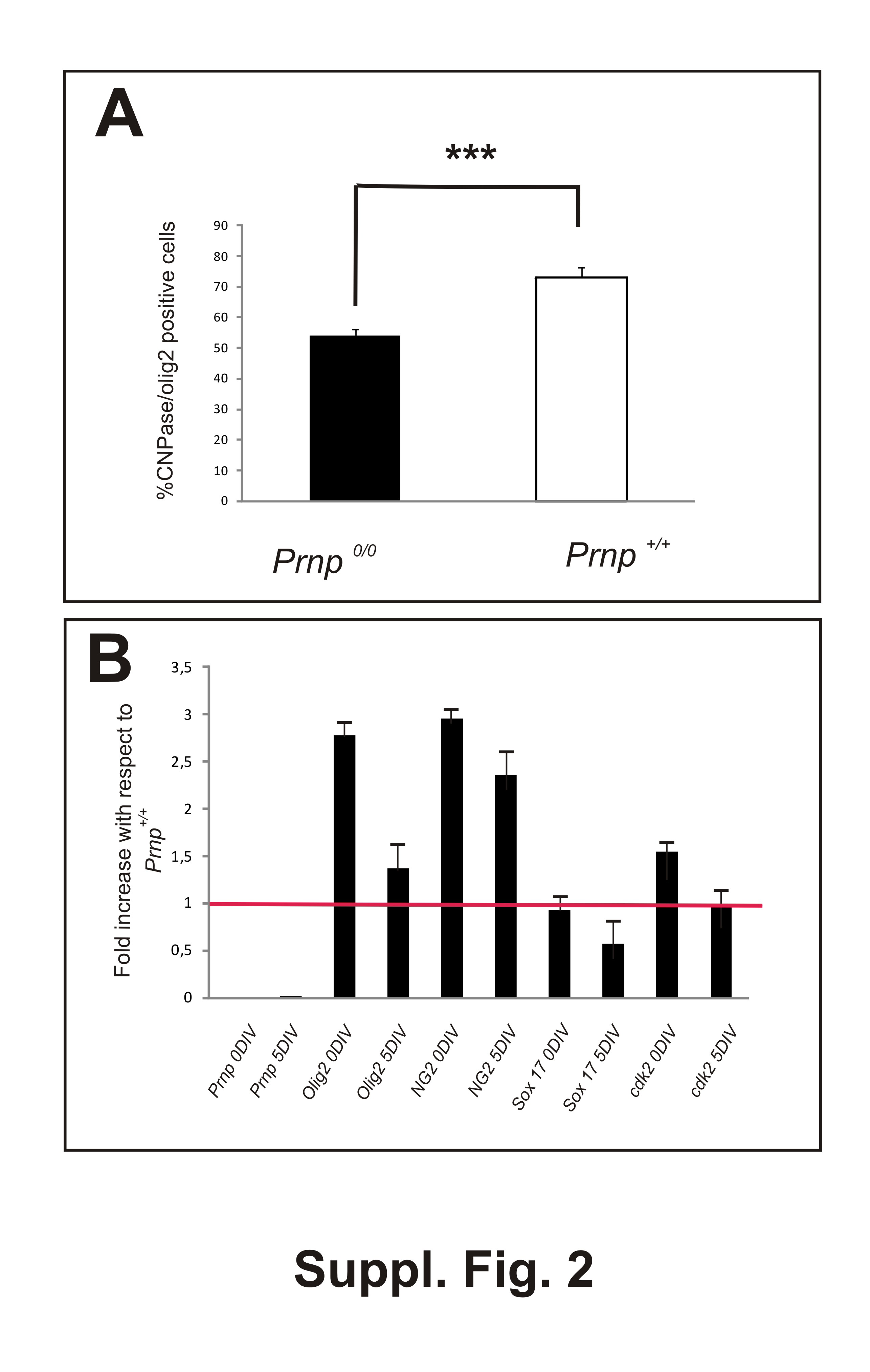

Supplement: Figure S2 — Prnp0/0 oligodendrocytes were less differentiated than the wild type oligodendrocytes in vitro . A) Histogram showed the quantification of the CNPase/Olig2 double labeled cells in cultures of isolated OPCs from Prnp0/0 and Prnp +/+ mice. Note that in Prnp0/0 cultures the proportion of mature oligodendrocytes was lower. Values represent the mean ± standard deviation, and the asterisks indicate statistical significance (P < 0.01, Student́s t-test). B) Histogram showing RT-qPCR analysis of RNA samples extracted from Prnp +/+ and Prnp0/0 purified oligodendrocytes after 0 and 5 DIV in SFM and without astrocytes. The data represent the induction of three independent experiments, with GAPDH used as the reference gene. (TIF) [file pone.0033872.s002.tif]

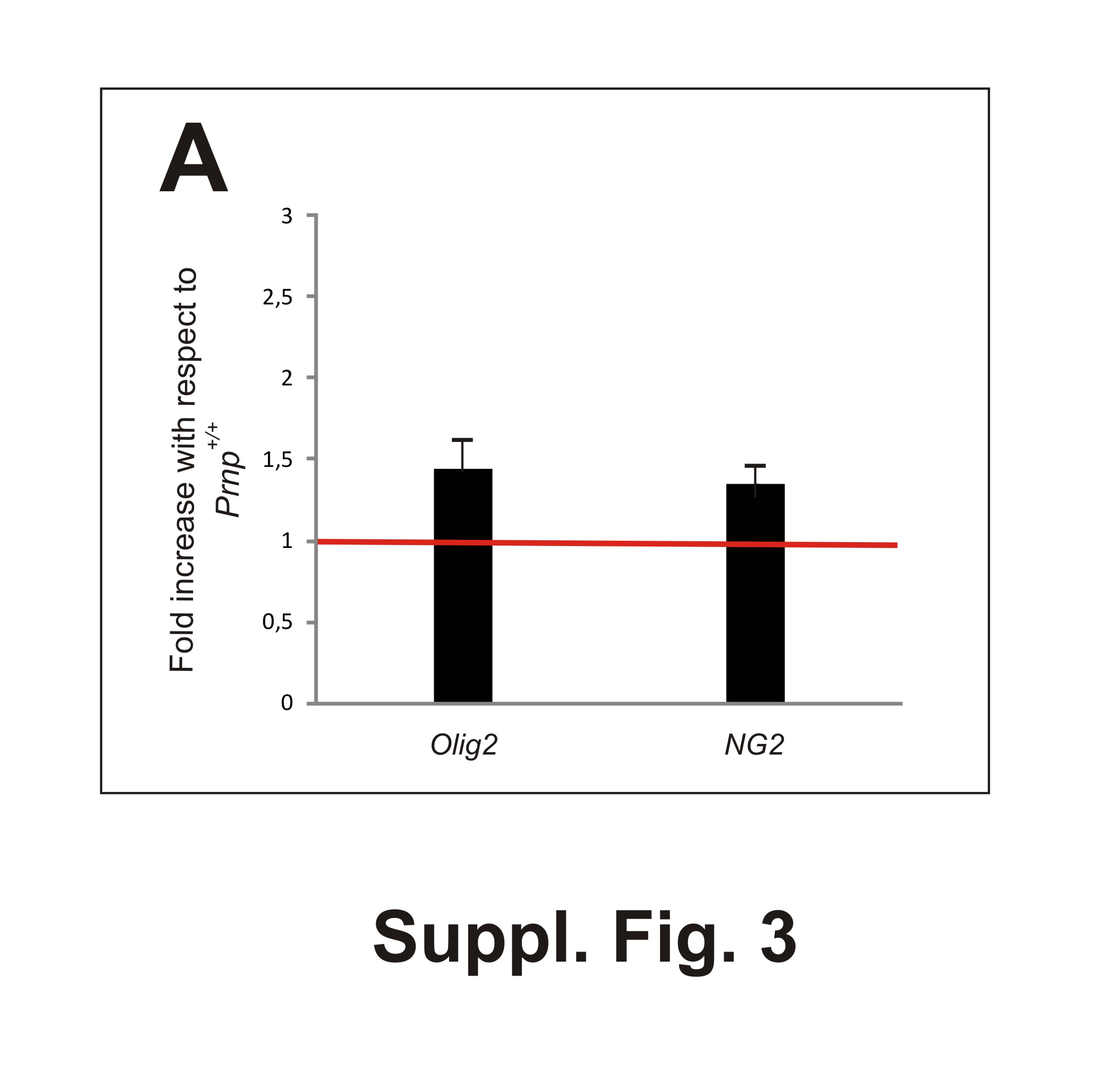

Supplement: Figure S3 — Prnp0/0 mice have increased levels of Olig2 and NG2 mRNA than Prnp +/+ mice. Histogram showing RT-qPCR analysis of RNA samples extracted from the adult Prnp +/+ and Prnp0/0 mouse cortex. Data represent the mean induction of three independent experiments in which GAPDH was used as the reference gene. (TIF) [file pone.0033872.s003.tif]

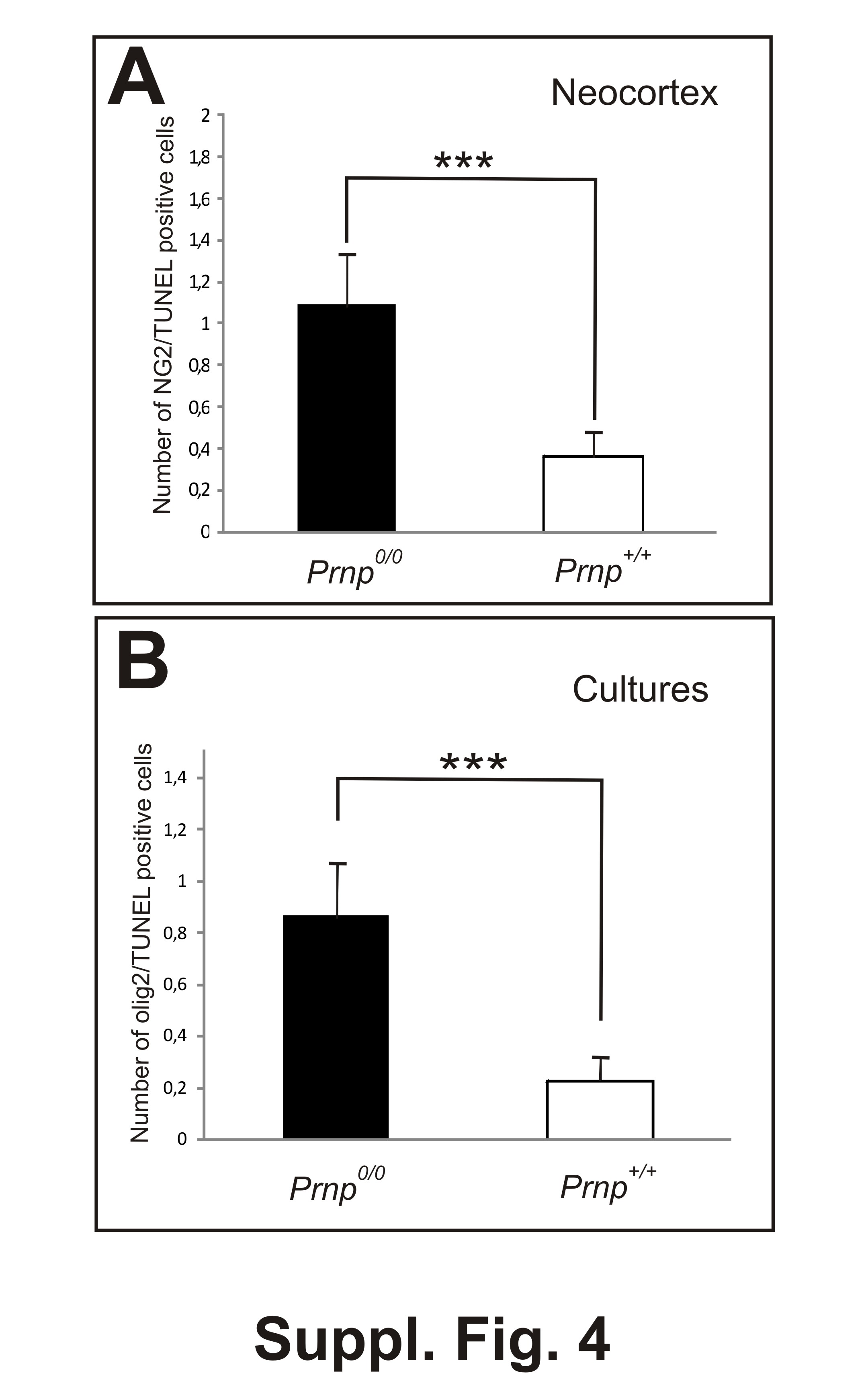

Supplement: Figure S4 — PrPc absence derived in OPCs less survival in vivo and in vitro . A) Histogram showed the number of NG2/TUNEL-positive cells in the neocortex of adult Prnp +/+ and Prnp0/0 mice. B) Histogram showed the number of double labeled Olig2/TUNEL cells in isolated oligodendrocytes derived from Prnp +/+ and Prnp0/0 cultures. In both cases there were more apoptotic oligodendrocytes in the absence of PrPc. Values in A and B represent the mean ± standard deviation, and the asterisks indicate statistical significance (P < 0.01, Student́s t-test). (TIF) [file pone.0033872.s004.tif]
